# Changes in metabolism but not myocellular signaling by training with CHO‐restriction in endurance athletes

**DOI:** 10.14814/phy2.13847

**Published:** 2018-09-02

**Authors:** Kasper D. Gejl, Kristian Vissing, Mette Hansen, Line Thams, Torben Rokkedal‐Lausch, Peter Plomgaard, Anne‐Kristine Meinild Lundby, Lars Nybo, Kurt Jensen, Hans‐Christer Holmberg, Niels Ørtenblad

**Affiliations:** ^1^ Department of Sports Science and Clinical Biomechanics University of Southern Denmark Odense Denmark; ^2^ Department of Public Health, Section for Sport Science Aarhus University Aarhus Denmark; ^3^ SMI Department of Health Science and Technology Faculty of Medicine Aalborg University Aalborg Denmark; ^4^ Department of Clinical Biochemistry Rigshospitalet Copenhagen Denmark; ^5^ The Centre of Inflammation and Metabolism Centre for Physical Activity Research Rigshospitalet University of Copenhagen Copenhagen Denmark; ^6^ Department of Nutrition, Exercise and Sports University of Copenhagen Copenhagen Denmark; ^7^ Swedish Winter Sports Research Centre Department of Health Sciences Mid Sweden University Östersund Sweden; ^8^ Swedish Olympic Committee Stockholm Sweden

**Keywords:** Cycling, endurance performance, fat oxidation, glycogen, train‐low

## Abstract

Carbohydrate (CHO) restricted training has been shown to increase the acute training response, whereas less is known about the acute effects after repeated CHO restricted training. On two occasions, the acute responses to CHO restriction were examined in endurance athletes. Study 1 examined cellular signaling and metabolic responses after seven training‐days including CHO manipulation (*n *=* *16). The protocol consisted of 1 h high‐intensity cycling, followed by 7 h recovery, and 2 h of moderate‐intensity exercise (120SS). Athletes were randomly assigned to low (LCHO: 80 g) or high (HCHO: 415 g) CHO during recovery and the 120SS. Study 2 examined unaccustomed exposure to the same training protocol (*n *=* *12). In Study 1, muscle biopsies were obtained at rest and 1 h after 120SS, and blood samples drawn during the 120SS. In Study 2, substrate oxidation and plasma glucagon were determined. In Study 1, plasma insulin and proinsulin C‐peptide were higher during the 120SS in HCHO compared to LCHO (insulin: 0 min: +37%; 60 min: +135%; 120 min: +357%, *P *=* *0.05; proinsulin C‐peptide: 0 min: +32%; 60 min: +52%; 120 min: +79%, *P *=* *0.02), whereas plasma cholesterol was higher in LCHO (+15–17%, *P *=* *0.03). Myocellular signaling did not differ between groups. p‐AMPK and p‐ACC were increased after 120SS (+35%, *P *=* *0.03; +59%, *P *=* *0.0004, respectively), with no alterations in p‐p38, p‐53, or p‐CREB. In Study 2, glucagon and fat oxidation were higher in LCHO compared to HCHO during the 120SS (+26–40%, *P *=* *0.03; +44‐76%, *P *=* *0.01 respectively). In conclusion, the clear respiratory and hematological effects of CHO restricted training were not translated into superior myocellular signaling after accustomization to CHO restriction.

## Introduction

Endurance training is a salient and powerful stimulus to induce both cellular and cardiovascular adaptations, and consequently to improve aerobic capacity and performance. Accordingly, numerous training strategies have been investigated in highly trained endurance athletes to optimize training outcomes and athletic performance, including restriction of carbohydrate (CHO) intake either before, during, or after exercise (Stellingwerff 2013; Hawley and Morton 2014). Thus, a deliberate change in the metabolism and substrate utilization by training with energy‐ or CHO restriction may be a strategy to improve the training response and endurance performance.

In terms of endurance performance, important myocellular adaptations imposed by training and/or dietary stimuli ultimately rely on accumulated exercise‐induced myocellular signaling for transcription of genes related to the metabolism (e.g., substrate utilization and mitochondrial biogenesis) (McGee and Hargreaves 2011). In this regard, metabolic perturbations incurred by single‐bout exercise activate signaling cascades related to mitochondrial adaptations and substrate utilization. These involve activation of 5′‐AMP‐activated protein kinase (AMPK) and p38 mitogen‐activated protein kinase (p38 MAPK) and down‐stream transcription factors such as peroxisome proliferator‐activated receptor gamma coactivator 1‐alpha (PGC1‐*α*), p53 and cAMP response element‐binding protein (CREB) (McGee and Hargreaves 2011; Impey et al. 2018). Since AMPK signaling is responsive to changes in AMP/ATP turnover, it is plausible that exercise under energy‐restricted circumstances may further accentuate AMPK signaling and enhance metabolically beneficial adaptations (Wojtaszewski et al. 2003; Yeo et al. 2010). In line with this, pre‐exercise energy restriction and muscle glycogen depletion have been shown to promote an acute up‐regulation of molecular markers associated with muscle oxidative capacity, mitochondrial biogenesis, and lipid oxidation when compared to a CHO‐enriched diet in both recreationally active individuals (Pilegaard et al. 2002, 2005; Cochran et al. 2010; Hawley et al. 2018) and endurance athletes (Wojtaszewski et al. 2003; Psilander et al. 2013; Lane et al. 2015). Importantly, previous investigations on acute responses in highly trained endurance athletes have entailed group differences in the timing of energy intake (Yeo et al. 2010; Jensen et al. 2015; Lane et al. 2015) or differences in the total energy intake between groups consuming low or high amounts of CHO (Psilander et al. 2013). Consequently, the acute regulatory effects of CHO restriction *per se* in highly trained endurance athletes deserve further attention.

In endurance‐trained individuals, acute effects of energy restriction or muscle glycogen depletion on myocellular signaling have typically been demonstrated in the unaccustomed state (Yeo et al. 2010; Psilander et al. 2013; Lane et al. 2015). However, the achievement of chronic measurable performance enhancing effects likely requires an accumulation of exercise‐induced metabolic perturbations during a prolonged period. In this regard, previous studies in highly trained individuals, regularly exposed to either periodized energy or CHO restriction during training, have not demonstrated a promotion of positive effects on endurance performance in comparison to a CHO enriched diet (Yeo et al. 2008; Hulston et al. 2010; Burke et al. 2017; Gejl et al. 2017). By contrast, moderately trained individuals have been shown to respond beneficially in some (Cochran et al. 2015; Marquet et al. 2016), but not all studies (De Bock et al. 2008; Morton et al. 2009; Van Proeyen et al. 2010). The lack of chronic effects in highly trained endurance athletes could likely be explained by a gradual decline in signaling sensitivity toward a familiar imposing stimuli. However, limited knowledge exists about the acute response to CHO restriction in the accustomed state, when applied repeatedly to a routine training schedule. Yet, such knowledge is particularly important, in order to establish practical recommendations for elite endurance athletes.

The purpose of the present study was to investigate the acute myocellular and metabolic response to CHO restricted training following preceding days with CHO restriction in highly trained endurance athletes. The use of an isocaloric design and a matched timing of calorie intake between groups ingesting low or high amounts of CHO during training and recovery, allowed us to evaluate the effects of CHO restriction *per se*. We hypothesized that with regular exposure to CHO restriction, the superior effects would be small or non‐existent compared to training in a CHO‐fed state. To further understand the initial findings of the study, we conducted a supportive study, investigating the substrate utilization and other metabolic parameters in response to the employed CHO manipulated training protocol.

## Methods

### Study overview

**Figure 1:**
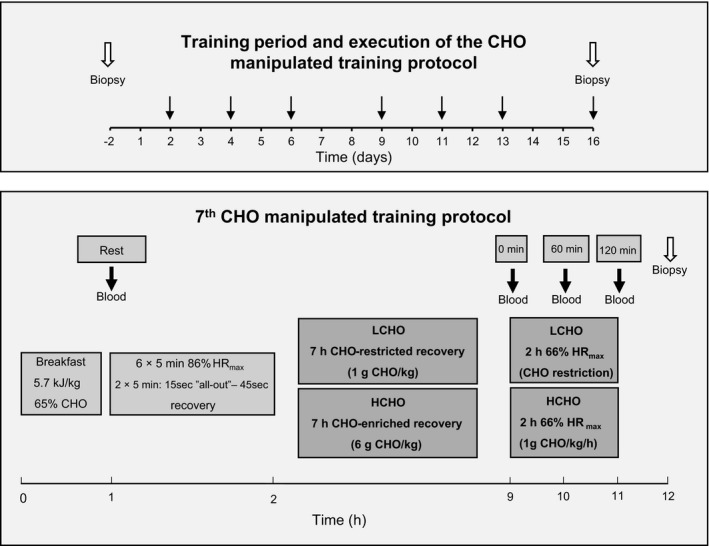
Study overview. *Upper section*: timing of the muscle biopsies before and acutely after performing the 7 days with CHO manipulation during the 16‐day training period (Study 1). Black arrows indicate timing of the days including the CHO manipulated training protocol and open arrows the obtainment of muscle biopsies. *Lower section*: detailed overview of the CHO manipulated training protocol and the acute muscle biopsy extraction. During the CHO manipulated training protocol, the groups received isocaloric diets containing different amounts of CHO and fat. The lower section further illustrates the timing of the standardized breakfast, the 1 h HIIT session and the period with CHO manipulation including both the post‐HIIT recovery and the 120SS (Study 1 and 2). The filled arrows indicate collection of blood samples prior to HIIT (Rest) as well as before (0 min), during (60 min), and after (120 min) the 120SS. The open arrow indicates the biopsy obtained 1 h after the 120SS.

Sixteen highly trained endurance athletes completed a 16‐day training period including seven days involving a CHO manipulated training protocol (Study 1) (Fig. 1). The protocol consisted of two training sessions and the acute myocellular response was investigated following the 7th of these training days. On days featuring the CHO manipulated training protocol, all subjects ingested a standardized CHO‐enriched breakfast and performed a 1 h high‐intensity interval training session (HIIT) one hour later. Thereafter, athletes recovered for 7 h while receiving isocaloric diets containing a low (LCHO) or high (HCHO) amount of CHO. This was followed by a 120‐min training session of moderate intensity (120SS), during which water or a CHO‐containing beverage were provided to the LCHO and HCHO groups respectively. The present study is a part of a larger investigation examining the effects of CHO periodization in highly trained endurance athletes (Gejl et al. 2017). For the present investigation, muscle biopsies were extracted from a subgroup of the athletes (*n* = 16).

To further support and understand findings of Study 1, a separate study was conducted one year later (Study 2). At the exact same time of the season, a similar group of highly trained endurance athletes conducted the protocol described above in an unaccustomed state. Here, we examined the respiratory response to the protocol (i.e., substrate utilization) while blood samples were obtained to determine plasma glucagon and blood glucose.

### Subjects, matching and ethical approval

Sixteen highly trained male triathletes and road cyclists were enrolled in the investigation (LCHO: 25 ± 2 years, 77 ± 2 kg, *V*O_2max_: 67 ± 2 mL O_2_ kg^−1^ min^−1^; HCHO: 26 ± 3 years, 74 ± 2 kg, *V*O_2max_: 70 ± 2 mL  O_2_ kg^−1^ min^−1^). All participants trained an average of at least 10 h per week throughout the year, demonstrated a *V*O_2max_ greater than 60 mL O_2_ kg^−1^ min^−1^, and had trained continuously at a high level for at least 2 years. Among the triathletes, eight were members of the Danish National Triathlon Team competing in international Olympic and sprint distance competitions (Olympic Games, World Triathlon Series, World Cups and Continental Cups), six participated in elite national competitions (Olympic, ½ ironman and ironman distances), while the remaining two competed at a lower level (½ ironman and ironman distances). The road cyclists possessed A‐licenses and competed at the elite national level.

To obtain equivalent groups for comparison, the participants were paired off on the basis of their primary sporting discipline (road cycling, Olympic‐distance triathlon, or long‐distance triathlon), training history and *V*O_2max_. During the 16‐day training period, the matched subjects in each pair were required to perform all training sessions together in an identical manner. This design ensured identical average training volumes and intensities between the two groups.

All subjects were fully informed of any potential risk associated with these experiments before providing verbal and written consents to participate. The Ethics Committee of Southern Denmark preapproved the study (Project‐ID S‐20150034), and all procedures adhered to the standards formulated in the *Declaration of Helsinki*.

### The CHO manipulated training protocol

The 16‐day training plans, designed by the Danish national triathlon coach, reflected the matched pairs′ routine training (i.e. differed between matched pairs) superimposed with the CHO manipulated training protocol three times per week. Accordingly, prior to the training period, one athlete from each pair was randomly assigned to LCHO and the other to HCHO (Fig. 1). Subjects avoided strenuous physical activity for 24 h prior to the days featuring CHO manipulation. The HIIT session consisted of 10 min of light warm‐up followed by six 5‐min intervals of cycling with an average target intensity of 85% HR_max_ and two 5‐min blocks of five 15‐s maximal sprints separated by 45s of light spinning to recruit type II fibers. Between each consecutive 5‐min intervals, 2 min of active recovery was allowed. This protocol is a modified version of the 8 × 5 min study design employed by Stepto et al. (2001), which reduced the muscle glycogen content in highly trained athletes by 50%. Seven hours after the HIIT session, each athlete performed the 120SS with a target intensity of 65% HR_max_ (Fig. 1).

Both training sessions were carried out on personal bikes by use of home‐trainers (Tacx Bushido Smart T2780, Wassenaar, Netherlands). As an indicator of training intensity, heart rate was monitored continuously (Polar Team 2, Polar Electro Oy, Kempele, Finland), and supervisors ensured that each subject maintained the predetermined intensity during the training sessions, with a gradual increase in the absolute training load (i.e., power output) from the 1st to the 7th seventh day containing the CHO manipulated training protocol (Gejl et al. 2017). One individual from each of the LCHO and HCHO groups was unable to attain the target workload of 85% HR_max_ during HIIT (80% and 83% HR_max_ respectively).

### Dietary manipulation

The timing of caloric intake and the total daily caloric intake were controlled and balanced in accordance with training volume and body mass on training days featuring the CHO manipulated training protocol. Each subject consumed a standard breakfast 60 min before the HIIT session (6.1 kcal kg bm^−1^, 65% CHO, 14% protein and 21% fat). During the 7 h recovery period, LCHO and HCHO were provided with isocaloric diets (2217–2495 kcal) containing 1 g and 6 g CHOkg bm^−1^ respectively. This corresponded to 81 ± 2 g and 414 ± 6 g CHO in LCHO and HCHO, respectively. Protein intake was similar in both groups throughout the day to prevent bias from this route for gluconeogenesis and to avoid manipulation with three macronutrients. Allergies and dislikes were taken into consideration at an individual level if alternatives held within the framework of energy content and macronutrients. During the 120SS, LCHO consumed water ad libitum, while HCHO consumed a beverage containing 1 g CHO kg bm^−1^ h^−1^. All food‐packages were designed by an experienced dietician using a suitable software program (Vitakost Aps, Denmark). The day before the seventh completion of the CHO manipulated training protocol, subjects consumed a standardized food‐package (42 kcal kg bm^−1^ day^−1^; 63% CHO, 15% protein, and 22% fat).

### *V*O_2max_ and incremental submaximal test

Measurements of *V*O_2_ have been described previously (Gejl et al. 2017). Briefly, a submaximal progressive step test and a maximal cycling test was conducted using an electronically braked ergometer (Schoberer Rad Messtechnik (SRM), 117 GmbH Julich, Germany). Using a mixing chamber system (CPX, Innovision, Glamsbjerg, Denmark) and based on the pulmonary ventilation and expiratory CO_2_ and O_2_ concentrations, both *V*O_2_ and *V*CO_2_ were determined. The submaximal step test involved 4‐min intervals with an initial workload of 135W and with increases of 35W every fourth minute until the RER value remained above 1.00 for one whole minute. During the maximal test, the initial 2‐min workload corresponded to the workload during the penultimate step of the submaximal test, after which it was increased by 25W every minute until exhaustion. The highest mean 30‐sec value for VO_2_ during the maximal test was defined as *V*O_2max_ while HR_max_ was defined as the highest heart rate observed during the test.

### Muscle biopsies & blood samples

Muscle biopsies of 100‐150 mg were obtained from the *m. vastus lateralis* portion of *m. quadriceps femoris* 2.5 weeks before (Rest) and 1 h after the 7th completion of the CHO manipulated training protocol (Acute) (Fig. 1). The timing of the acute biopsy was based on previous studies demonstrating upregulations of the investigated targets 0 to 3 h post exercise (Sriwijitkamol et al. 2007; Camera et al. 2010; Bartlett et al. 2013). The resting biopsy was obtained after consumption of a standardized diet during the preceding 24 h (Gejl et al. 2017). Muscle biopsies were obtained using 5 mm Bergström needles. The procedure for extraction of muscle tissue was identical at both time points and conducted by using the percutaneous needle biopsy technique as previously described (Bergstrom 1975). Biopsies were obtained randomly from the right and left thighs. Part of the biopsy was immediately frozen in liquid nitrogen and stored at −80°C for subsequent analysis of metabolite content as well as immunoblotting.

Blood samples were obtained before the high‐intensity morning session (Rest) as well as before (0 min), halfway through (60 min) and after (120 min) the 120SS (Fig. 1). Samples of venous blood were drawn from the antecubital vein and deposited in clean glass tubes (5 mL for insulin and proinsulin C‐peptide; Study 1 only) or anticoagulant single‐use containers; Li‐Heparin (4 mL for Triglycerides, LDL, HDL and Cholesterol, Study 1 only), K2‐EDTA (2 mL for blood glucose; both studies) or K3‐EDTA (5 mL for glucagon; Study 2 only). After collection, the blood samples were immediately stored at 4°C and within 10 min, centrifuged at 2000 g at 4°C for 10 min. Aliquots of plasma and serum were stored at −80°C. Total plasma cholesterol, LDL, HDL, triglycerides, insulin, and c‐peptide were measured using a Cobas^®^ 8000 Modular analyzer (Roche Diagnostics, Basal, Switzerland). Plasma glucagon concentrations were determined by a glucagon RIA assay (catalog number GL‐32K, Millipore, Billerica, USA) using a 1470 Wizard gamma counter (Perkin Elmer, Wallac, USA). Changes in hemoglobin concentration were used to correct for the effects of dehydration, assuming that the total content of hemoglobin remained unchanged.

### Muscle glycogen

Muscle glycogen content was determined spectrophotometrically (Beckman DU 650) (Passonneau and Lowry 1993). Freeze‐dried muscle tissue (1.5 mg) was boiled in 0.5 mL 1M HCL for 150 min before it was quickly cooled, whirl‐mixed, and centrifuged at 3500*g* for 10 min at 4°C. 40 *μ*L of boiled muscle sample and 1 mL of reagent solution containing Tris‐buffer (1mol/L), distilled water, ATP (100 mmol/L), MgCl_2_ (1mol/L), NADP^+^ (100 mmol/L), and G‐6‐PDH were mixed before the process was initiated by adding 10 *μ*L of diluted hexokinase. Absorbance was recorded for 60 min before the glycogen content was calculated. Muscle glycogen content is expressed as mmol·kg dw^−1^. Data on muscle glycogen has previously been reported in a companion manuscript involving 26 athletes engaged in a 4‐week training study (Gejl et al. 2017). In the present acute study, glycogen data from 16 athletes is presented and will be used to interprete the findings resulting from the CHO manipulated training protocol.

### Immunoblotting

20 mg of frozen muscle tissue was freeze‐dried and subsequently homogenized, separated, and electroblotted as previously described (Rahbek et al. 2015). The following primary antibodies were purchased from Cell Signalling Technology (Danvers, MA) and utilized as follows; phospho‐specific AMPK^Thr172^ (cat # 2531, conc. 1:1000 in 5% BSA), phospho‐specific P38 MAPK^Thr180/Tyr182^ (cat # 4511, conc. 1:1000 in 5% BSA), phospho‐specific ACC^Ser79^ (cat # 3661, conc. 1:1000 in 5% BSA), phospho‐specific CREB^Ser133^ (cat # 9198, conc. 1:1000 in 5% BSA), phospho‐specific p53^Ser15^ (cat # 9286, conc. 1:1000 in 5% skim milk). With regards to secondary antibodies, for all targets except p53, membranes were then incubated for 1 h with horseradish peroxidase‐conjugated goat anti‐rabbit (Cat # 2054, Santa Cruz,TX, USA) and utilized as follows: phospho‐specific AMPK^Thr172^ (conc. 1:3000 in 1% BSA), phosphospecific p38 MAPK^Thr180/Tyr182^ (conc. 1:5000 in 1% BSA), phosphospecific ACC^Ser79^ (conc. 1:5000 in 1% BSA), phosphospecific CREB^Ser133^ (conc. 1:10.000 in 1% BSA). Specifically for phospho‐specific p53, the membrane was incubated for 1 h with horseradish peroxidase‐conjugated goat anti‐mouse (Cat # 2055, Santa Cruz,TX, USA) secondary antibody (concentration: 1:5000 in TBST and 1% skim milk). Proteins were visualized by chemiluminiscence (Thermo Scientific, MA, USA) and quantified with a UVP imaging system (UVP, CA, USA). Precision Plus Protein All Blue standards were used as markers of molecular weight (Bio‐Rad, CA). Values derived for quantification of immunoblotting for each protein target were normalized to the total amount of protein loaded for each sample, using the Stain Free Technology approach previously described (Gilda and Gomes 2013; Gurtler et al. 2013).

## Study 2

### Substrate utilization during the 120SS

To further investigate findings from the study described above, a second study was conducted on a separate occasion, but at the same time of the season. Here, the effects of the CHO manipulated training protocol on substrate utilization and plasma glucagon concentrations were determined. 12 highly trained male triathletes (LCHO: 24 ± 3 years, 77 ± 5 kg, *V*O_2max_: 71 ± 6 mL O_2_ kg^−1^ min^−1^; HCHO: 28 ± 8 years, 78 ± 4 kg, *V*O_2max_: 69 ± 8 mL O_2_ kg^−1^ min^−1^), of which 10 were also included in the initial study, replicated the CHO manipulated training protocol with all procedures being equal to the first study. During the 120SS, the *V*CO_2_:*V*O_2_ ratio (RER) and fat oxidation rate were calculated regularly (0–10 min, 30–40 min, 60–70 min, 90–100 min and 115–120 min) using the same equipment as described above. The fat oxidation rate was calculated based on the stoichiometric equation by Frayn (1983): fat oxidation rate = (1.67 × *V*O_2_) – (1.67 × *V*CO_2_), with the assumption that the urinary nitrogen excretion was negligible. Respiratory data was obtained from 10 of 12 athletes due to technical problems. Additionally, blood samples were obtained at the same time‐points as in the first study, and analyzed for blood glucose and plasma glucagon.

### Statistical analyses

Statistical analyses of the effects of CHO manipulation were carried out utilizing a two‐way ANOVA with repeated measures (group vs. time) and a Sidak post‐hoc test, with correction for multiple comparison (Graph Pad Prism 6.07). Because of one broken sample during the homogenization process (subject from HCHO) and a destroyed membrane for some targets (LCHO: ACC and CREB), a few measurements were not included in the analyses (*see* legend Fig. 3). Values are expressed as means ± SD, and the normalized data from immunoblotting was log‐transformed before being analyzed by two‐way ANOVA and shown as geometric means ± back‐transformed SD. Statistical comparison of findings from the Study 1 and 2 was performed by a student's *t* test. *P* values ≤ 0.05 denotes statistical significance. Two comparable studies, including 6–7 individuals in each group (Yeo et al. 2010; Lane et al. 2015), have previously demonstrated significant acute effects of CHO manipulation on AMPK and ACC phosphorylation. Based on the p‐AMPK results of those studies, with differences between groups of 2.0 and 1.0 (AU) and SD′s of 1.3 and 0.5 in the two studies, respectively, a minimum sample size of 6‐8 subjects in each group was calculated as needed to attain a power of 0.80. A similar sample size (*n *=* *6) was calculated as needed for p‐ACC (Lane et al. 2015). For fat oxidation, using a mean difference of 0.22 g min^−1^ and a SD of 0.12, five subjects were necessary in each group to attain a power of 0.80.

## Results

### Subjects characteristics and workload during the CHO manipulated training protocol

The athletes in LCHO and HCHO were similar with respect to *V*O_2max_, body mass, height, age, and training history (*see “Methods”)*.

The first six 5‐min intervals of HIIT were conducted at 86 ± 4% of HR_max_ in LCHO and 85 ± 5% HR_max_ in HCHO with no difference between groups. The steady state intensity during the last three minutes, was 89 ± 4% HR_max_ in both groups. During the 120SS, the workload was 66 ± 2% HR_max_ in both groups.

### Glucose regulation during the CHO manipulated training protocol

**Figure 2:**
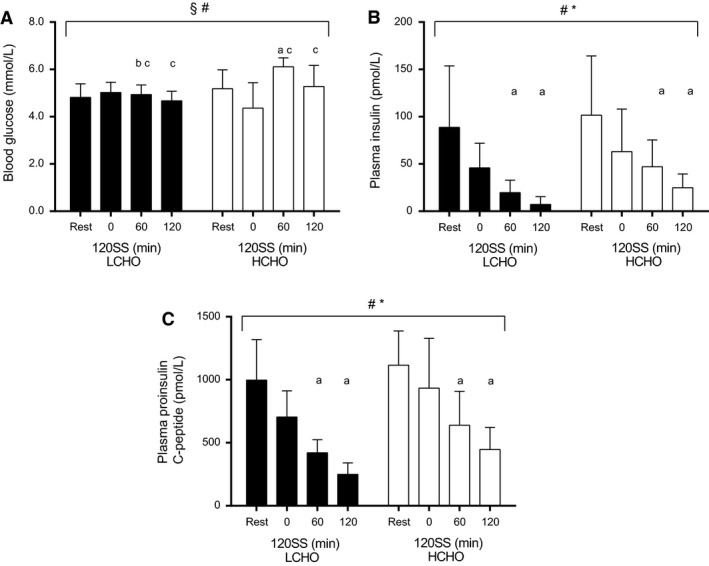
Mean blood glucose (A), plasma insulin (B) and proinsulin C‐peptide concentrations (C) throughout the 120SS at 65% HR_max_ in LCHO (filled bars, *n *=* *8) and HCHO (open bars, *n *=* *8) during the 7th completion of the CHO manipulated training protocol (Study 1). § *P *<* *0.05 for the group x time interaction, # *P *<* *0.05 for the overall time‐effect, * *P *<* *0.05 for the overall difference between LCHO and HCHO, ^a^ *P *<* *0.05 in comparison to Rest; ^b^ *P *<* *0.05 in comparison to HCHO at the same time point; ^c^ *P *<* *0.05 in comparison to identical time point during the unaccustomed conduction of the CHO manipulated training protocol (Study 2).

The blood glucose time course differed between groups (group x time interaction: *P *=* *0.0008), primarily due to the pronounced elevation of blood glucose in the HCHO group after 60 min of the 120SS (*P *<* *0.01), with no changes in the LCHO group (Fig. 2A). While the blood glucose remained stable in the LCHO group, it fluctuated during the CHO manipulated training protocol in the HCHO group, eventually being similar to Rest after the 120SS (5.3 ± 0.9 mmol L^−1^).

Although plasma levels of insulin and proinsulin C‐peptide were reduced in both groups throughout the CHO manipulated training protocol (*P *<* *0.0001) (Fig. 2B and C), these levels were generally higher in the HCHO group compared to the LCHO group (insulin: 0 min: + 37%; 60 min: +135%; 120 min: +357%, *P *=* *0.05; proinsulin C‐peptide: 0 min: + 32%; 60 min: +52%; 120 min: +79%, *P *=* *0.02).

Muscle glycogen was reduced to a similar extent in both groups acutely after the CHO manipulated training protocol (−31 ± 21%; 648 to 431 mmol kg dw^−1^; −39 ± 12%; 652 to 396 mmol kg dw^−1^ in LCHO and HCHO, respectively, *P *<* *0.0001) (Gejl et al. 2017).

### Myocellular signaling following the CHO manipulated training protocol

**Figure 3:**
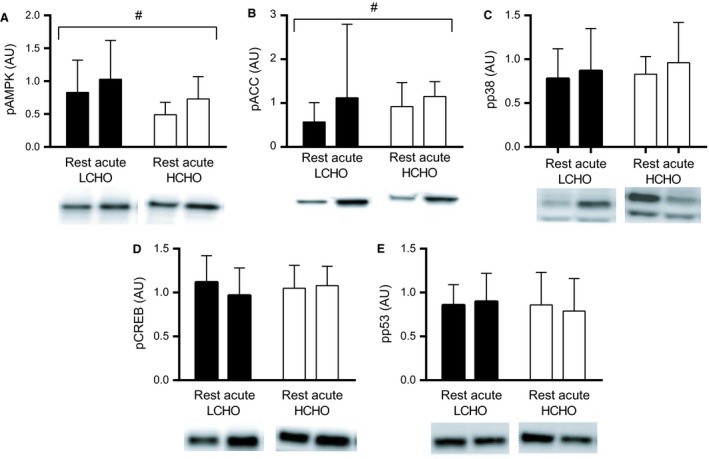
Mean p‐AMPK (A), p‐ACC (B), p‐CREB (C), p‐p38 (D) and p‐p53 (E) at rest and 1 h following the 120SS at 65% HR_max_ in LCHO (filled bars) and HCHO (open bars) after the 7th completion of the CHO manipulated training protocol (Study 1). Representative blots are presented below each target. Note that the lower band in figure 3C represents p‐p38. AMPK:* n *=* *8 and 7; ACC:* n *=* *7 and 7; CREB:* n *=* *7 and 7; p38: *n *=* *8 and 7; p53: *n *=* *8 and 7 in LCHO and HCHO respectively. # *P *<* *0.05 for the overall time‐effect.

Following the CHO manipulated training protocol, the muscle level of p‐AMPK was higher than at Rest (*P *=* *0.03), with no difference between the LCHO and HCHO group (Fig. 3A). Similarly, an overall time effect was evident with respect to the phosphorylation of ACC (*P *=* *0.0004). Neither p‐p38, p‐53, nor p‐CREB exhibited changes after the 120SS in neither the LCHO nor the HCHO group (Fig. 3C‐E).

### Plasma cholesterol and triglyceride during the CHO manipulated training protocol

Resting plasma levels of LDL, HDL, cholesterol, and triglyceride were identical in both groups (Table 1). LDL levels were higher in the LCHO group compared to the HCHO group during the 120SS (29–30%; *P *=* *0.008), as were the cholesterol levels (16%; *P *=* *0.03) (Table 1).

**Table 1:** Plasma lipid profiles at rest and during the 120SS

		Rest	0 min	60 min	120 min
LDL (mmol L^−1^)	Low CHO	2.36 ± 0.49	2.19 ± 0.27^b^	2.20 ± 0.26^b^	2.26 ± 0.29b#*
	High CHO	2.05 ± 0.52	1.69 ± 0.29^a^	1.71 ± 0.29^a^	1.74 ± 0.26a#
HDL (mmol L^−1^)	Low CHO	1.37 ± 0.19	1.53 ± 0.25^a^	1.51 ± 0.24^a^	1.56 ± 0.25a#
	High CHO	1.46 ± 0.31	1.54 ± 0.39	1.54 ± 0.38	1.56 ± 0.38a#
Cholesterol (mmol L^−1^)	Low CHO	4.11 ± 0.58	4.30 ± 0.27^b^	4.26 ± 0.32^b^	4.29 ± 0.34b§
	High CHO	3.90 ± 0.63	3.72 ± 0.47	3.69 ± 0.45	3.68 ± 0.44
Triglycerides (mmol L^−1^)	Low CHO	1.20 ± 0.65	1.68 ± 0.64^a^	1.43 ± 0.52	1.25 ± 0.35#
	High CHO	1.22 ± 0.39	1.40 ± 0.57	1.30 ± 0.52	1.08 ± 0.34#

^#^ *P* < 0.05 for the overall time effect; *P < 0.05 for the overall group difference; ^§^ *P* < 0.05 for the group × time interaction; ^a^ *P* < 0.05 in comparison to Rest; ^b^ *P* < 0.05 in comparison to HCHO at same time‐point.

No group differences in the levels of HDL and triglycerides were observed during the 120SS. HDL was elevated in both groups after the 120SS compared to Rest (LCHO: +14%; HCHO: +7%, *P *<* *0.05) but also at 0 min and 60 min in the LCHO group (+11% and +12%, respectively; *P *<* *0.001) (Table 1). Before the 120SS, triglyceride levels were elevated solely in the LCHO group compared to Rest (+54%, *P *<* *0.001), while these levels declined from 0 min to 120 min of the 120SS in both groups (LCHO: −22%; HCHO: −20%, *P *<* *0.05) (Table 1).

## Study 2

### Substrate utilization during the CHO manipulated training protocol

**Figure 4:**
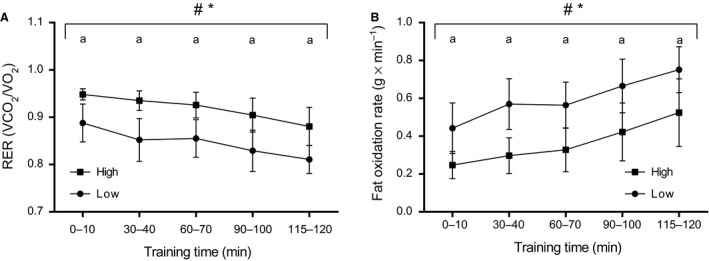
Mean RER values (A) and fat oxidation rates (B) throughout the 120SS at 65% HR_max_ in LCHO (circles, *n *=* *5) and HCHO (squares, *n *=* *5) during unaccustomed exposure to the CHO manipulated training protocol (Study 2). Respiratory data from two subjects are missing due to technical issues during the 120SS. # *P *<* *0.05 for the overall time‐effect; * *P *<* *0.05 for the overall difference between LCHO and HCHO; ^a^ *P *<* *0.05 for the difference between LCHO and HCHO.

Workload during Study 2 was similar to that observed in Study 1 (HIIT: 86 ± 4% HR_max_ and 86 ± 4% HR_max_ in LCHO and HCHO, respectively; 120SS: 66 ± 2% HR_max_ in both groups). The RER during the 120SS was consistently lower for the LCHO compared to the HCHO group (LCHO: 0.89 ± 0.04 to 0.81 ± 0.03 vs. HCHO: 0.95 ± 0.01 to 0.88 ± 0.04, *P *=* *0.008), and declined in both groups (*P *<* *0.0001) (Fig. 4A). Accordingly, fat oxidation increased gradually in both groups (*P *<* *0.0001), but was markedly higher in the LCHO group throughout the session (LCHO: 0.44 ± 0.13 to 0.75 ± 0.12 g min^−1^ vs. HCHO: 0.25 ± 0.07 to 0.52 ± 0.18 g min^−1^, *P *=* *0.01) (Fig. 4B).

### Blood glucose and glucagon during the CHO manipulation day

**Figure 5:**
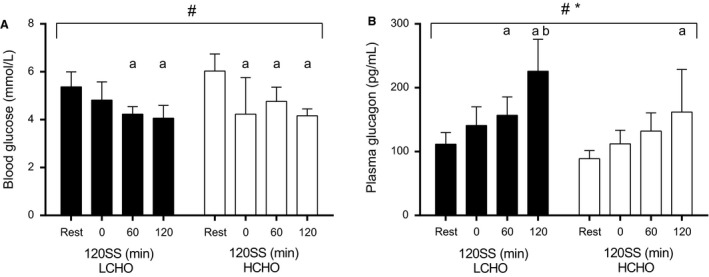
Mean blood glucose (A) and plasma glucagon concentrations (B) throughout completion of the 120SS at 65% HR_max_ in LCHO (filled bars, *n *=* *6) and HCHO (open bars, *n *=* *6) during unaccustomed exposure to the CHO manipulated training protocol (Study 2). # *P *<* *0.05 for the overall time effect; *P <* *0.05 for the overall difference between LCHO and HCHO; ^a^ *P *<* *0.05 in comparison to Rest; ^b^ *P *<* *0.05 in comparison to HCHO.

During Study 2, blood glucose was reduced to a similar extent in both groups throughout the 120SS when compared to Rest (*P *=* *0.0001) (Fig. 5A). After the 120SS, blood glucose was 4.1 ± 0.4 and 4.2 ± 0.3 mmol L^−1^ in the LCHO and HCHO group, respectively, and thus significantly lower compared to Rest in both groups (*P *<* *0.01).

Plasma levels of glucagon rose gradually throughout the 120SS in both groups when compared to Rest (*P *<* *0.0001) (Fig. 5B). However, glucagon was generally higher in the LCHO group (*P *=* *0.03), being 40% higher after the 120SS (LCHO: 226 ± 42 pg mL^−1^ vs. HCHO: 162 ± 71 pg mL^−1^, *P *<* *0.05).

The blood glucose responses from both Study 1 and 2 were compared in order to investigate possible adaptations with repeated training. Interestingly, changes in blood glucose were different between the unaccustomed conduction of the CHO manipulated training protocol (Study 2) and the seventh conduction of the same protocol (Study 1) (Figs. 2A and 5A). During the 7th exposure to the protocol, blood glucose was 17% (*P *=* *0.02) and 15% (*P *=* *0.04) higher after 60 min and 120 min of the 120SS with the CHO restriction, while it was 28% (*P *=* *0.006) and 27% (*P *=* *0.02) higher with the CHO enriched diet (Figs. 2A and 5A). Thus, repeating the same CHO manipulated training protocol seems to prevent perturbations in blood glucose.

## Discussion

To our knowledge, this is the first study to investigate the acute response to multiple days of CHO restricted training (i.e., without energy restriction), on substrate utilizatoin and myocellular signaling in highly trained endurance athletes. The results suggest that repeated exposure to CHO restriction during exercise, without caloric restriction, does not enhance the acute training response in highly trained athletes. The present lack of group differences in p‐AMPK, p‐ACC, p‐p53, p‐CREB and p‐p38 MAPK could likely be explained by the identical muscle glycogen levels that existed despite the ingestion of diets varying markedly in their CHO contents (Impey et al. 2016).

### Myocellular signaling

The ATP turnover during a single bout of endurance exercise can be expected to stimulate myocellular AMPK signaling for promoting metabolic adaptations, such as mitochondrial biogenesis (McGee and Hargreaves 2011), and this may be augmented under conditions with low carbohydrate availability (Pilegaard et al. 2002, 2005; Wojtaszewski et al. 2003; Cochran et al. 2010; Yeo et al. 2010; Psilander et al. 2013; Lane et al. 2015). Previously, changes in myocellular signaling by CHO or energy restriction have been demonstrated after exposure to a double‐session training protocol in the unaccustomed state in endurance‐trained individuals (Yeo et al. 2010; Psilander et al. 2013; Jensen et al. 2015; Lane et al. 2015). Yet, to achieve chronic measurable performance‐enhancing effects, the increased response by CHO or energy restriction must likely be imposed repeatedly throughout a prolonged period of training. The 7th completion of the present CHO manipulated training protocol produced an increase in AMPK phosphorylation and downstream phosphorylation of ACC to promote LCFA entry to the mitochondria, but importantly, these changes were similar between groups and thus not enlarged by CHO restriction (Vissing et al. 2008, 2013). On the other hand, in the accustomed state, we did not observe increased phosphorylation of p38 MAPK and the downstream signaling substrates CREB and p53 by the training protocol in neither LCHO nor HCHO. These findings are largely similar to those observed by Yeo et al. (2010) immediately after performance of HIIT training with low and high muscle glycogen, respectively. Although the level of p‐AMPK following exercise was greatest in the energy restricted group in that study, it was also elevated in the group with high muscle glycogen and moreover no changes emerged in downstream targets. By contrast, Lane and coworkers reported that a “sleep‐low” approach accentuated exercise‐induced signaling for metabolic, but not mitochondrial, adaptations in highly trained athletes (Lane et al. 2015). It should be appreciated that the protocol used Lane and colleagues differs from our study in several respects (e.g. dietary protocol, CHO vs. energy periodization, and time course of measurements). A likely explanation for the discrepancy in the myocellular responses in comparison with other studies in highly trained endurance athletes (Wojtaszewski et al. 2003; Psilander et al. 2013; Lane et al. 2015) is likely the absence of severe muscle glycogen depletion in the present study, perhaps as a result of high resting muscle glycogen levels and adaptation to the imposed training stimulus (*see* discussion *below*) (Impey et al. 2016).

Based on earlier findings by Yu and colleagues and later Coffey and colleagues, the absence of significant changes in phosphorylation of AMPK, p38 MAPK, CREB, and p53 may also be partly explained by highly trained athletes exhibiting low sensitivity to a type of exercise stimuli to which they are highly familiar (Yu et al. 2003; Coffey et al. 2006). To this end, the observed lack of pronounced effect in LCHO on myocellular protein signaling may partly relate to the fact that these measures were evaluated in a familiarized condition with respect to CHO restriction. In line with this, unaccustomed exposure to HIIT followed by a prolonged moderate‐intensity afternoon session in Study 2, elicited a decline in blood glucose levels during the afternoon training while this response was attenuated as athletes were accustomed to the CHO manipulated training protocol in both LCHO and HCHO in Study 1. These results suggest that the preceding 16 days of training either decreased muscle glucose uptake or that hepatic glucose output was increased due to higher hepatic glycogenesis. This further indicates that by repeating the same training protocol it becomes increasingly difficult to achieve metabolic disturbances that may be necessary to augment the cellular training response in highly trained athletes, despite increases in the absolute workload (Gejl et al. 2017).

The present results show that the CHO manipulation strategy was indeed effective in promoting metabolic group differences in highly trained athletes. Important regulators of lipolysis (i.e., insulin and proinsulin C‐peptide) were significantly lower in the LCHO group compared to the HCHO group, while glucagon, plasma cholesterol, and LDL were significantly higher in LCHO compared to HCHO during the 120SS. In line with this, the fat oxidation rate was markedly higher during the 120SS in LCHO compared to HCHO, which is in accordance with previous observations in highly trained individuals (Wojtaszewski et al. 2003; Lane et al. 2015). Based on the above mentioned metabolic group differences it is somewhat surprising that muscle glycogen availability remained identical between groups, even after the 120SS.

### CHO and energy periodization in highly trained endurance athletes

Several strategies involving periodized CHO or energy restriction have been evaluated in highly trained endurance athletes during the past decade. Two early training studies in highly trained endurance athletes applied the promising design developed by Hansen et al. (2005) to cycling exercise. These studies compared training every day under conditions with restored muscle glycogen levels with training twice every other day(Yeo et al. 2008; Hulston et al. 2010). Although the design complicates discrimination between effects of training with reduced muscle glycogen *per se* and potential effects of different training distributions and intensities, these findings indicated that optimization of the manipulation protocol could lead to superimposing effects on performance in highly trained athletes. A recent 3‐week training study in moderately trained triathletes investigated a protocol consisting of a HIIT session in a glycogen replenished state in the afternoon followed by CHO and energy restriction overnight (“sleep‐low”) and eventually a moderate‐intensity session in the morning (Marquet et al. 2016). Ten kilometer running performance was improved (≈3%) in the “sleep‐low” group of this study. However, this improvement could, at least in part, be attributed to a decrease in body mass as suggested by Mettler et al. (2017).

By matching training load and intensity, as well as calorie intake and the timing hereof, we recently presented the effects of implementing the present CHO restriction protocol 11 times during a 4‐week training period on endurance performance and myocellular adaptations in a group of highly trained athletes (Gejl et al. 2017). As presented here, the CHO manipulated training protocol induced significant acute differences in substrate utilization as well as TG, LDL, and cholesterol levels between groups during training. This furthermore indicates that plasma NEFA levels were also acutely elevated in the LCHO group, which has previously been shown to increase mitochondrial biogenesis in the skeletal muscle (Garcia‐Roves et al. 2007). However, these clear acute metabolic differences were not translated into different training effects in neither resting muscle glycogen levels, enzyme activity, myocellular signaling, nor performance between groups (Gejl et al. 2017). It could be argued that repeating the exact same training protocol will eventually lead to a plateau in the acute adaptations, despite a gradual increase in the absolute work load (Gejl et al. 2017). The aforementioned difference in blood glucose response between the two present investigations, with a maintenance of blood glucose after preceding exposure to the CHO manipulation protocol, supports this notion. However, exposure to a variety of different CHO‐ and energy restriction strategies also failed to demonstrate superimposing training effects in a recent comprehensive 3‐week study in highly trained race walkers (Burke et al. 2017).

### Practical implications

The general lack of superimposing effects of CHO periodization in “real‐life” training studies with highly trained individuals could be explained by high post exercise muscle glycogen levels, and the small acute net degradation of glycogen in LCHO in the present study suggests that vigorous “train‐low” protocols are needed to repeatedly reduce muscle glycogen to very low levels (≤250 mmol kg dw^−1^). We have previously demonstrated that 4 h of prolonged CHO‐restricted cycling exercise at ≈75% of HR_max_ depletes muscle glycogen in highly trained triathletes (from 699 ± 24 mmol kg dw^−1^ to 225 ± 28 mmol kg dw^−1^), while Psilander and coworkers used a sophisticated protocol consisting of both HIIT and continuous work to deplete muscle glycogen level in a group of athletes (to ≈170 mmol kg dw^−1^) (Psilander et al. 2013; Gejl et al. 2014). However, demanding protocols as described above are difficult to complete multiple times per week in the schedule of elite endurance athletes if other training stimuli should not be compromised, and the implementation must be carefully planned as suggested by Impey and colleagues (Impey et al. 2018).

The present findings emphasize the importance of discriminating between CHO restriction *per se* and strategies involving overall periodized energy restriction. From the high muscle glycogen levels observed after the present “train‐low” protocol, CHO restriction *per se* does not seem to be an appropriate method in highly trained endurance athletes and overall calorie restriction may be necessary to reduce muscle glycogen sufficiently. Calorie restriction once a week or once every other week could be an appropriate compromise, leading to superimposing effects on performance. However, this needs further investigation in a design reflecting the “real life” of elite endurance athletes.

Finally, all CHO manipulating approaches imply pros and cons. Here, we used an isocaloric approach to investigate the effects of manipulating macronutrient composition *per se* on the acute training response, implying that fat intake was increased in LCHO because of the reduced CHO intake. Thus, we cannot exclude that the increased fat intake may have affected the acute training response. Moreover, while the dietary conditions were similar prior to and during the HIIT session, the higher fat oxidation in LCHO during the 120SS likely affected the HR‐power output relationship (Cole et al. 2014). Thus, while metabolic demands (i.e., % of *V*O_2max_) were likely similar between groups, power output was probably slightly smaller in the LCHO group during the 120SS.

In conclusion, the present findings demonstrate that the acute cellular response after consecutive CHO restricted training sessions is not improved compared with the response following training with a high CHO intake. Accordingly, similar myocellular responses emerged despite clear metabolic differences after the ingestion of low and high amounts of CHO, respectively. The lack of superior effects of CHO restriction *per se* on the activation of drivers of potential performance‐enhancing adaptations may be explained by high postexercise muscle glycogen levels. Future studies must provide effective and practical applications of CHO periodization strategies to legitimize its relevance in highly trained endurance athletes.

## Conflict of Interest

The authors report no conflict of interest and the results of the present study do not constitute endorsement by ACSM. We declare that the results of the study are presented clearly, honestly and without fabrication, falsification, or inappropriate data manipulation.
